# Palatal Lengthening With Double-Opposing Buccal Flaps for Velopharyngeal Insufficiency

**Published:** 2017-08-18

**Authors:** Paige C. Goote, Nicholas S. Adams, Robert J. Mann

**Affiliations:** ^a^Michigan State University College of Human Medicine, Grand Rapids, Mich; ^b^GRMEP/Michigan State University Plastic Surgery Residency, Grand Rapids, Mich; ^c^Helen DeVos Children's Hospital Pediatric Plastic and Craniofacial Surgery, Grand Rapids, Mich

**Keywords:** palate lengthening, cleft palate, velopharyngeal dysfunction, buccal flap, speech surgery

## DESCRIPTION

**Figure 1:**
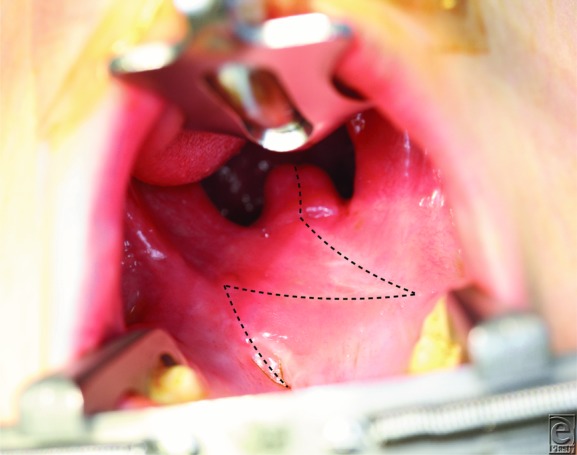
Hard and soft palates of a 5-year-old boy with a history of Furlow double-opposing Z-plasty palatoplasty (dashed line). Note the short palatal length and nonanatomical location of the velum relative to the posterior pharyngeal wall.

A 5-year old boy presented to the pediatric plastic surgery clinic for hypernasal speech. He was born with a cleft palate and underwent repair at 11 months of age. A short velum was noted (Fig 1), so he underwent palatal lengthening with double-opposing buccal flaps. Postoperatively, his hypernasality was corrected.

## QUESTIONS

1. **What is velopharyngeal dysfunction (VPD)?**
2. **How is velopharyngeal insufficiency (VPI) diagnosed?**
3. **What treatment options are available for VPI?**
4. **What is the palatal lengthening with double-opposing buccal flaps procedure?**

## DISCUSSION

Velopharyngeal dysfunction is defined as the inability of the velopharyngeal valve (VPV) to close properly.^1^ It manifests as altered speech and nasal regurgitation. Velopharyngeal dysfunction can be classified into 3 main categories based on the underlying cause. Velopharyngeal incompetence is due to a neurological or neuromuscular cause leading to impaired function of the VPV despite normal anatomy. Velopharyngeal mislearning is a less common form of VPD, in which function and anatomy of the velopharyngeal (VP) mechanism are intact, but the patient has mislearned how to produce certain speech sounds accurately. Velopharyngeal insufficiency represents an inadequate closure of the VPV due to anatomical or structural abnormalities. It can develop secondary to surgical intervention, congenital malformations, or trauma. The most common congenital causes of VPI include cleft palate and velopharyngeal disproportion. Surgical procedures such as palatoplasty, tumor resection, and adenotonsillectomy can also result in VPI.^2^

Velopharyngeal insufficiency can be diagnosed through auditory and instrumental methods, with both methods frequently utilized to obtain an accurate diagnosis.^3^ Perceptual speech evaluation allows articulation, resonance, nasal airway emission, and voice to be assessed.^4^^,^^5^ Video fluoroscopy and nasendoscopy are the most common imaging modalities used in the diagnosis of VPI.^5^ These methods provide direct, dynamic images of the anatomy and function of the VPV in vivo.^3^ They can assess closure patterns, gaps, and anatomical variations, which are useful in both identifying the etiology of dysfunction and surgical planning.^2^

Nonsurgical treatment options for VPI include speech therapy and prosthetics. Speech therapy remains the first-line treatment of nearly all patients with VPI.^3^ As an isolated therapy, it has the highest success in patients with mild insufficiency. When surgical intervention is necessary, speech therapy almost always accompanies the procedure. Obturators and prostheses are most useful in patients in whom surgery is not an option.^2^ The goal of surgical intervention is to narrow the nasopharynx and restore VPV competence without causing hyponasality or upper airway obstruction.^4^ The Furlow: double-opposing Z-plasty palatoplasty reconstructs the levator sling by repositioning the posterior myomucosal flap. This returns the levator musculature to the appropriate horizontal physiological position in addition to lengthening the velum.^3^^,^^5^ In a posterior pharyngeal flap, the soft palate is surgically adhered to a superiorly based flap from the posterior pharyngeal wall, creating a static structure that reduces the size of the velopharyngeal opening in the sagittal plane. This repair may be more successful in VPD with intact lateral pharyngeal wall motion. The sphincter pharyngoplasty utilizes bilateral palatopharyngeal flaps from the posterolateral pharyngeal walls. They are rotated posterior and medially and connected with the posterior pharyngeal wall with a joining incision. This procedure may be better for patients with poor VPV motion in the coronal plane.^2^^,^^3^

**Figure 3:**
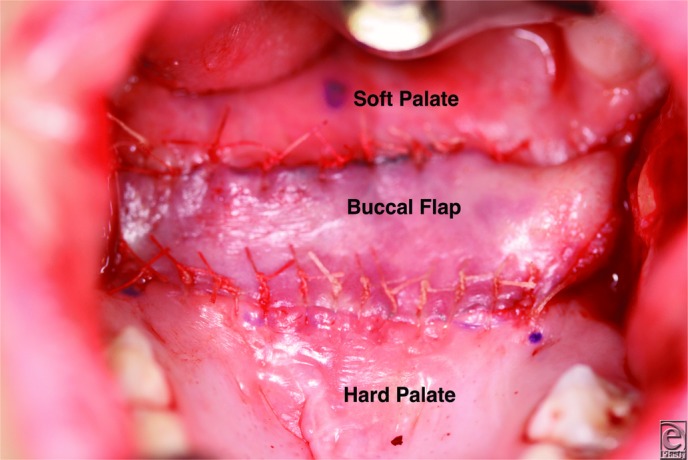
After releasing the soft palate from the hard palate, the bilateral buccal flaps are interposed in the resultant space. One flap reconstructs the nasal mucosa and the other is opposed to reconstruct the oral mucosa. The inset buccal flap is seen here from the oral side. Note the soft palate in a more posterior, anatomical position.

**Figure 4:**
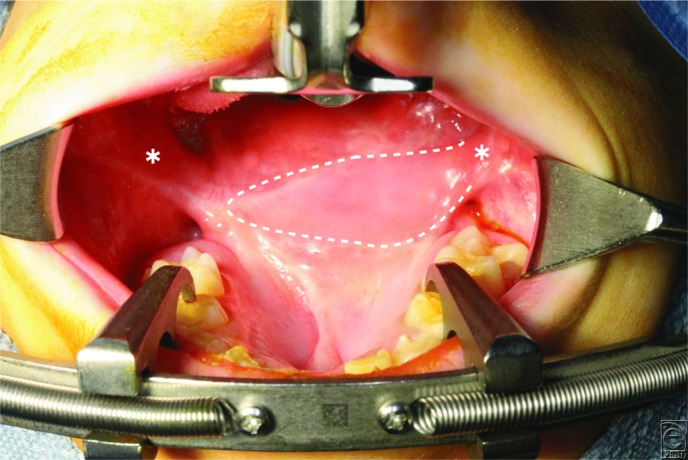
Twenty-two months postoperatively. The palate is completely healed with pliable mucosal tissue interposed between the hard and soft palates (dashed line). Note the pedicles of the bilateral buccal flaps near the retromolar trigone. These will be divided and inset at a later procedure.

Palatal lengthening with bilateral opposed buccal flaps (Figs 2*a* and 2*b*) is designed to correct VPI by allowing the velum to move posterior and improving the closure of the VP sphincter.^6^ In this procedure, the velum is released from the hard palate with a transverse incision. Abnormal muscle attachments are released, and the soft palate falls posteriorly into a more anatomical position. Bilateral buccal myomucosal flaps based at the retromolar trigone are then raised to fill the soft tissue gap between the hard and soft palates (Figs 3 and 4). The 2 flaps are opposed together, with the mucosa of the flaps reconstructing the nasal and oral sides of the palatal defect. This approach avoids surgical alteration of the velar muscles, decreasing potential scarring and leading to excellent speech outcomes.^6^

## CONCLUSION

Velopharyngeal insufficiency is commonly seen in patients with cleft palate. Many techniques have been described to improve VP function and speech. The palatal lengthening procedure has been shown to be a safe procedure with minimal donor site morbidity and no postoperative sleep apnea.

## Figures and Tables

**Figure 2:**
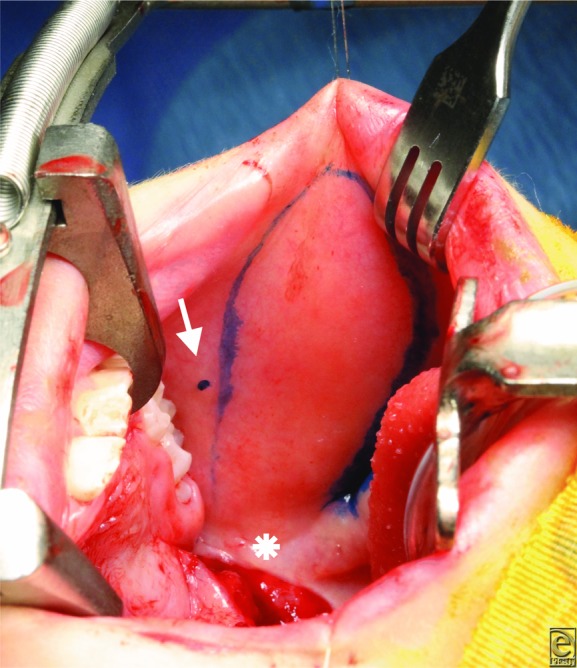
(a) The buccal flap is marked just inferior to the opening of the parotid duct (arrow). The pedicle is based at the retromolar trigone (asterisk).

**Figure 2:**
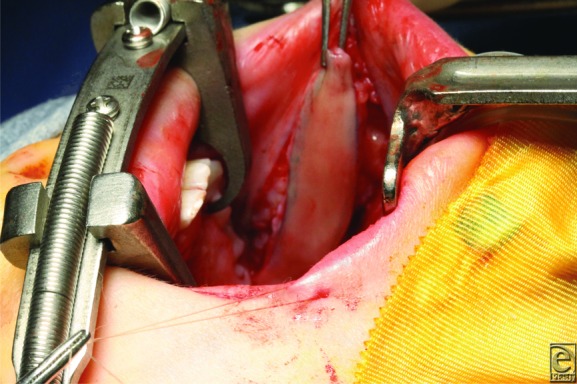
(b) Elevated buccal flap. A majority of buccinator muscle is included within the flap.

## References

[1] Loney RW, Bloem TJ (1987). Velopharyngeal dysfunction: recommendations for use of nomenclature. Cleft Palate J..

[2] Kirschner RE, Baylis AL, Rodriguez ED, Losee JE, Neligan PC (2012). Velopharyngeal dysfunction. Plastic Surgery: Craniofacial, Head and Neck Surgery Pediatric Plastic Surgery.

[3] Paniagua LM, Signorini AV, da Costa SS, Collares MVM, Dornelles S (2013). Velopharyngeal dysfunction: a systematic review of major instrumental and auditory-perceptual assessments. Int Arch Otorhinolaryngol.

[4] Fisher DM, Sommerlad BC (2011). Cleft lip, cleft palate, and velopharyngeal insufficiency. Plast Reconstr Surg.

[5] Gart MS, Gosain AK (2014). Diagnosis and management of velopharyngeal insufficiency following cleft palate repair. J Cleft Lip Palate Craniofac Anomalies.

[6] Mann RJ, Neaman KC, Armstrong SD, Ebner B, Bajnrauh R, Naum S (2011). The double-opposing buccal flap procedure for palatal lengthening. Plast Reconstr Surg.

